# The Efficacy and Safety of Brolucizumab for the Treatment of nAMD: A Systematic Review and Meta-Analysis

**DOI:** 10.3389/fphar.2022.890732

**Published:** 2022-05-13

**Authors:** Junlan Chuan, Lianqiao Liu, Yumei Feng, Mengdan Wang, Gang Li, Qin Lv

**Affiliations:** ^1^ Department of Pharmacy, Sichuan Academy of Medical Sciences and Sichuan Provincial People’s Hospital, School of Medicine, University of Electronic Science and Technology of China, Chengdu, China; ^2^ Personalized Drug Therapy Key Laboratory of Sichuan Province, School of Medicine, University of Electronic Science and Technology of China, Chengdu, China; ^3^ Department of Pharmacy, Mianyang People’s Hospital, Mianyang, China; ^4^ Traffic Hospital of Sichuan Province, Chengdu, China; ^5^ Department of Respiratory and Critical Care Medicine, Sichuan Academy of Medical Science and Sichuan Provincial People’s Hospital, School of Medicine, University of Electronic Science and Technology of China, Chengdu, China

**Keywords:** neovascular age-related macular degeneration, brolucizumab, efficacy, safety, meta-analysis

## Abstract

**Introduction:** As demonstrated in pivotal clinical trials, brolucizumab can be used to treat neovascular age-related macular degeneration (nAMD) because it antagonizes vascular endothelial growth factor (VEGF) in the vitreous. However, brolucizumab may cause retinal vasculitis obliterans in the presence of inflammation in the eyes. In the present study, a meta-analysis of randomized controlled trials (RCTs) was conducted to evaluate the efficacy and safety of brolucizumab.

**Methods:** ClinicTrail.gov., Embase, Cochrane Library, and PubMed were retrieved from inception until 31 December 2021 for RCTs assessing the efficacy and safety of brolucizumab. Changes in best corrected visual acuity (BCVA) and central sub-field thickness (CSFT) and incidence of adverse events, serious adverse events, and serious ocular adverse events were extracted from eligible RCTs. A meta-analysis was performed using RevMan 5.4.1.

**Results:** A total of six RCTs with 3,574 participants were finally involved in this meta-analysis. The changes of best corrected visual acuity (BCVA) showed no statistically significant difference between the brolucizumab-treated group and aflibercept-treated group. Brolucizumab induced higher central sub-field thickness (CSFT) reduction than the control agent (aflibercept). The incidence of adverse events was similar between the brolucizumab group and control group (OR 0.63, 95% CI 0.37 to 1.08, *p* = 0.09), and brolucizumab caused fewer serious adverse events (OR 0.78, 95% CI 0.63 to 0.95, *p* = 0.01). However, brolucizumab could lead to more serious ocular adverse events than Lucentis and aflibercept (OR 2.15, 95% CI 1.11 to 4.16, *p* = 0.02).

**Conclusion:** Brolucizumab was non-inferior to other anti-VEGF agents in improving BCVA and decreasing CSFT. But it caused more serious ocular adverse events which is worthy of special attention by ophthalmologists.

## 1 Introduction

Age-related macular degeneration (AMD), characterized by macular atrophy, scarring, and chronic exudation, is the dominant cause of irreversible permanent vision damage among individuals more than 55 years old in the developed countries (Fleckenstein et al., 2021; Georges et al., 2014). According to AMD’s international classification and grading system (Bird et al., 1995), the disease is divided into early AMD, characterized by drusen, and late AMD. Late AMD can be further classified into dry and wet forms, with geographic atrophy and choroidal neovascularization, respectively, and the wet form being more severe and causing more vision loss (Nowak. 2006; Stahl. 2020). Age-related accumulation of metabolic wastes, chronic aberrant inflammation, and oxidative stress in the elderly contributes to the disturbance of equilibrium between angiogenic and antiangiogenic cytokines (Ohno-Matsui et al., 2001; Colak et al., 2012). Choroidal neovascularization is then triggered by elevated expression of vascular endothelial growth factor (VEGF) (Tong et al., 2006).

VEGF is involved in the development of blood vessels, playing a crucial role in stimulating abnormal blood vessel growth and neovascularization (Yeo et al., 2019). Anti-VEGF agents, inhibiting VEGF activity by binding to VEGF and preventing its biological effects, were confirmed to limit visual loss in wet AMD patients and prevent progression of the disease (Gillies et al., 2019; Tano and Ohji, 2010). Currently, available VEGF antagonists mainly involve brolucizumab, conbercept, aflibercept, bevacizumab, ranibizumab, and pegaptanib (Hussain et al., 2021), of which ranibizumab, aflibercept, and bevacizumab have become three first-line anti-VEGF drugs for nAMD (Luu et al., 2021).

Brolucizumab, a monoclonal antibody that reduces neovascularization by binding to VEGF-A (2020, Tadayoni et al., 2021), was first approved in 2019 (Markham, 2019). It has a single-chain antibody fragment and low molecular weight, with significant structural differences from other VEGF inhibitors, making brolucizumab more durable and the next generation of therapeutically available anti-VEGF (Rahman and Singer, 2020). Brolucizumab is an intraocular injection formulation with a recommended dose of 6 mg every 4 weeks for the first 3 months, then changing to every 8–12 weeks. Several studies (NCT01304693, NCT01796964, NCT02507388, NCT02307682, NCT02434328, and NCT03386474) have investigated brolucizumab’s efficacy and safety for wet AMD (ClinicalTrials.gov. 2013; ClinicalTrials.gov, 2015; ClinicalTrials.gov, 2016; ClinicalTrials.gov, 2017; ClinicalTrials.gov, 2020; ClinicalTrials.gov, 2021). Two large studies, HAWK and HARRIER, demonstrated non-inferiority of brolucizumab and an overall safety profile similar to that of aflibercept (Zhang et al., 2021). However, given the different control drugs, dosages, and administration durations of the aforementioned randomized controlled trials, a comprehensive meta-analysis was valuable to evaluate the subtle differences between efficacy and safety of brolucizumab and other anti-VEGF drugs. Therefore, this meta-analysis of existing RCTs was conducted to provide synthesized results about the efficacy and adverse events of brolucizumab.

## 2 Methods

This meta-analysis was conducted with full reference to the guidelines of Preferred Reporting Items for Systematic Reviews and Meta-Analysis (PRISMA), and its introduction, methods, results, and discussion were in compliance with PRISMA regulations. RCTs evaluating brolucizumab’s efficacy and safety from Clinictrail.gov., Embase, Cochrane Library, and PubMed databases, published before 31 December 2021, were included in the study with no language restriction.

### 2.1 Search Strategy

We searched through several databases using a combination of subject and free words. Taking PubMed as an example, the following steps were performed: use subject headings derived from medical subject heading (MeSH) terms such as Macular Degeneration and brolucizumab; add their respective free words; and then use RCTs to restrict to do the search. Retrieved studies from various databases were manually reviewed to determine articles for inclusion.

### 2.2 Study Selection

Studies that met the following criteria were included in the meta-analysis: first, RCTs with at least one experimental arm (brolucizumab) and one control arm are included. Second, the subjects should be patients with nAMD. Finally, findings including any of the number of adverse events, severe adverse events, ocular severe adverse events, and the mean changes from baseline in BCVA/CSFT are necessary. This standard includes the main points of the PICOS principles. There are no restrictions on brolucizumab dosing intervals, total duration of treatment, or comparator. Reviews, case reports, expert opinions, pharmacoeconomic reports, pharmacokinetic studies, and preclinical studies were excluded. All information was synthesized when the study was a multi-publication one.

### 2.3 Data Extraction and Quality Assessment

Two researchers reviewed the retrieved articles independently, first looking at the titles and abstracts of the articles and then performing full-text screening to distinguish articles that met the inclusion criteria. Disagreements were resolved by a third investigator. A spreadsheet was built to collect data, which includes research leader or organization, publication time, study length, study group and number of patients, injection intervals and doses, total course of treatment, basic characteristics, mean change of BCVA/CSFT levels with SD, and the number of adverse events, severe adverse events, and severe ocular adverse events in experiment and control groups. Two reviewers independently extracted information, and the third author resolved discrepancies when necessary. The reviewers in this meta-analysis were blinded to authors, institutions, and journals of the studies when they extracted the data. The Cochrane Collaboration Tool (RevMan 5.4) was used to assess the study quality by bias analysis. Then, another two investigators evaluated the included studies separately, and the other resolved differences.

### 2.4 Statistical Analysis

The mean difference (MD) and 95% confidence interval (CI) in BCVA/CSFT change from baseline, as continuous variables, would be reported as outcomes in this study. The incidence of adverse events, severe adverse events, and severe ocular adverse events were shown in this study as relative risk (RR) and 95% CI. The incidence of adverse events and serious adverse events was calculated by dividing the total number of patients with adverse events or serious adverse events, while the incidence of serious ocular adverse events was calculated by dividing the total number of patients with the total number of events. When encountering more than one experimental group (brolucizumab), the efficacy and safety index data of several groups were merged and then compared with the control group (Georges et al., 2014). Heterogeneity between experiments was assessed using Cochran’s Q and *I* ^2^ values. When *p* < 0.1, *I* ^2^ > 75%, it means high heterogeneity between trials; when *p* < 0.1, 50% < *I* ^2^ < 75%, it means moderate heterogeneity; and when *p* > 0.1, *I* ^2^ < 50%, it indicates low heterogeneity. In statistics, moderate and high heterogeneities were modeled with random effects; low heterogeneity was modeled with fixed effects. In addition, sensitivity analysis was conducted to investigate potential heterogeneity sources. In addition, a funnel plot was used to detect publication bias; a *p*-value less than 0.05 indicated statistical significance.

## 3 Results

### 3.1 Study Selection

**FIGURE 1:**
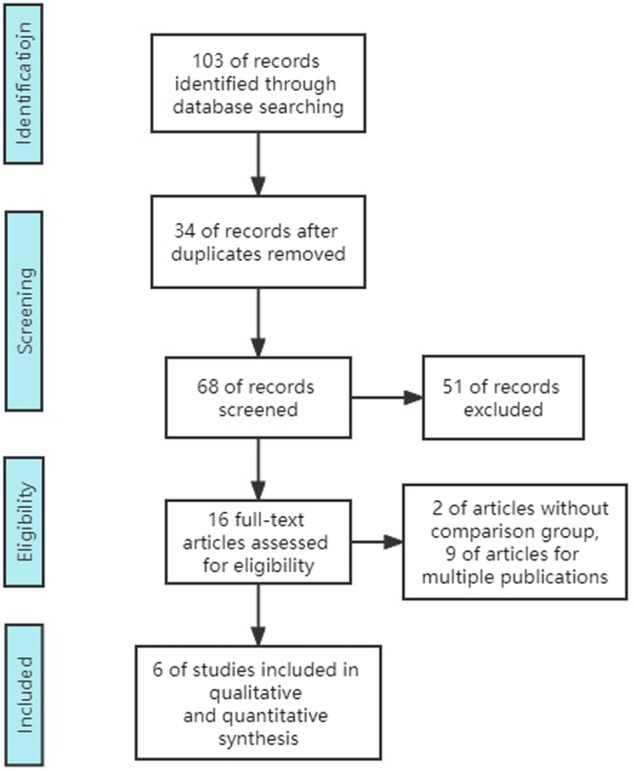
PRISMA flow diagram of eligible studies.

Although there were 102 studies retrieved in total, only 6 studies including 6 RCTs (3,574 participants) were included. Excluded studies consisted of 34 duplicates, 16 reviews, 15 unrelated studies, 2 single-arm research studies, 8 multi-publication studies, 9 conference or editorial articles, and 12 articles without results (no outcome data) (Figure 1).

### 3.2 Study Characteristics

**FIGURE 2:**
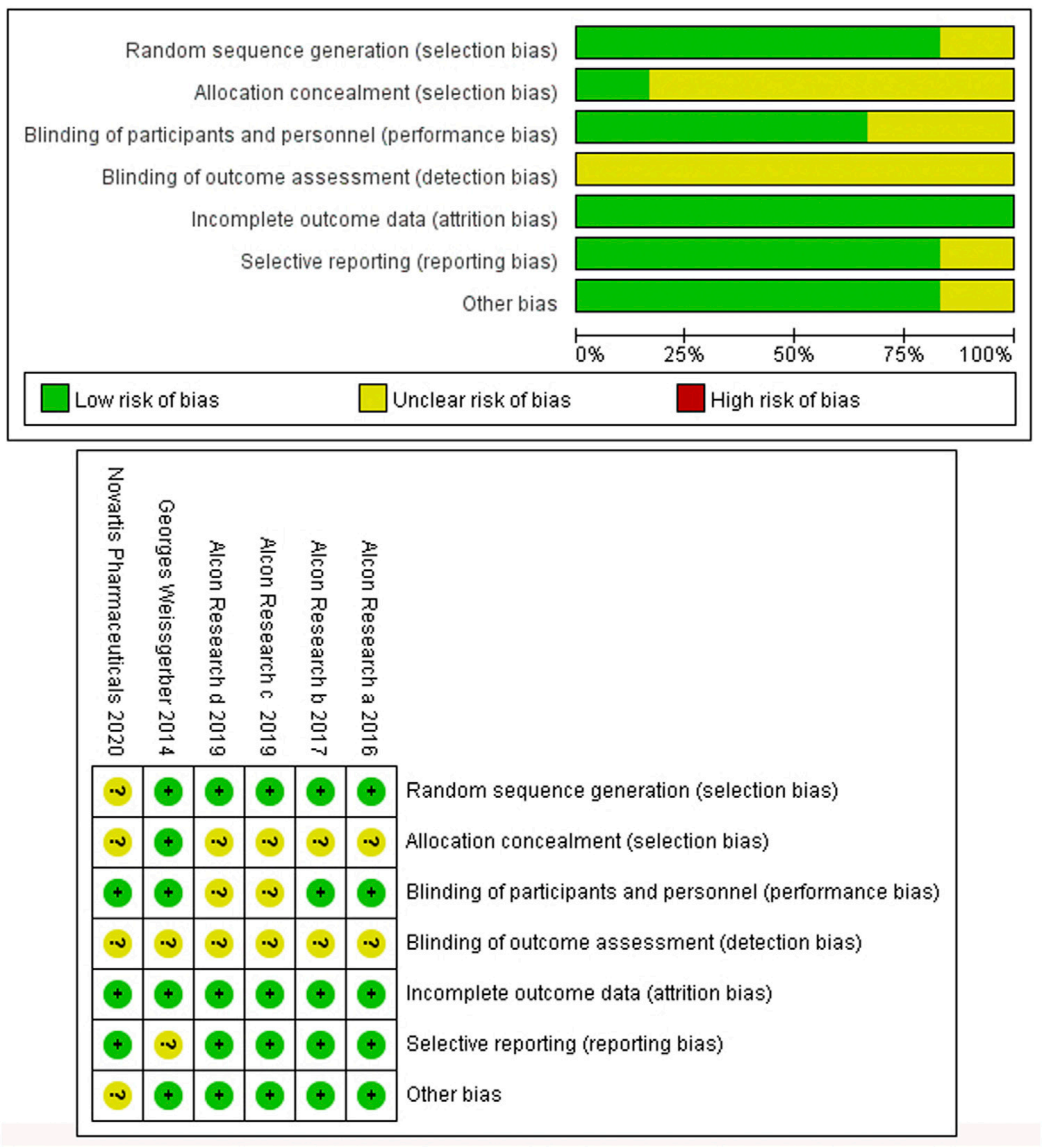
Risk of bias graph **(A)** and Risk of bias summary **(B)**.

Six studies involving six RCTs published between 2014 and 2020 were included. One RCT was a phase 1 trial, three were phase 2 trials, and three were phase 3 trials. The six RCTs lasted an average of 696 days, and 55.9% of the subjects were male. Table 1 shows the detailed characteristics. Table 2 shows baseline subjects’ characteristics and BCVA and CSFT characteristics. The risk of bias detection showed that no high risk of bias was presented in all included RCTs, whereas the unclear risk of bias was mainly concentrated in blinding of outcome assessment and allocation concealment. When judging performance bias, disagreement about the risk grade occurred between two investigators (LL and MW) because HAWK and HARRIER just mentioned double-blindness without explaining how to achieve blindness. In the end, the third (JC) author judged the disagreement to be an unclear risk according to the Cochrane Collaboration rules. Incomplete reporting data were classified as low risk because research personnel had little loss to follow-up and complete pre-specified endpoints were reported (Figure 2).

**TABLE 1:** Characteristics of the included six RCTs.

Research leader or organization	Year	Trial type	NCT number	Phase	Duration (days)	Intervention	Subject	Primary endpoint
Georges Weissgerber	2014	RCT	NCT01304693	1,2	882	Brolucizumab	Patients with nAMD	^f^
Alcon Research^a^	2016	RCT	NCT01796964	2	518	Brolucizumab	Patients with nAMD	^e^ ^,^ ^f^
Alcon Research^b^	2017	RCT	NCT02507388	2	51	Brolucizumab	Patients with nAMD	^f^
Alcon Research^c^	2019	RCT	NCT02307682	3	1775	Brolucizumab	Patients with nAMD	^e^ ^,^ ^f^
Alcon Research^d^	2019	RCT	NCT02434328	3	1,048	Brolucizumab	Patients with nAMD	^e^ ^,^ ^f^
Novartis Pharmaceuticals^g^	2020	RCT	NCT03386474	3	235	Brolucizumab	Patients with nAMD	^e^ ^,^ ^f^

a Efficacy and Safety Study of ESBA1008 versus EYLEA^®^.

b Safety and Pharmacokinetics of RTH258 in Subjects with Age-Related Macular Degeneration.

c Efficacy and Safety of RTH258 versus Aflibercept—Study 1 (HAWK).

d Efficacy and Safety of RTH258 versus Aflibercept—Study 2 (HARRIER).

e Study of Safety and Efficacy of Brolucizumab 6 mg Drug Product Intended for Commercialization in Patients with nAMD.

f The incidence of severe adverse events and severe ocular adverse events.

g Best corrected visual acuity (BCVA) change from baseline by visit in 1, 3, 6, 9, and 12 month.

**TABLE 2:** Baseline characteristics of subjects of six RCTs.

Study	Subgroup within the study	Case, n	Male, n (%)	Age, mean (standard deviation)	BCVA mean (standard deviation)	CSFT mean (standard deviation)
(Georges et al., 2014)	Lucentis, 0.5 mg, 1 IVT	61	28 (45.9%)	77.8 (8.1)	Not provided	Not provided
	Brolucizumab dose A (0.5 mg), 1 IVT	10	4 (40.0%)	75.9 (6.9)	Not provided	Not provided
	Brolucizumab dose B (3.0 mg), 1 IVT	35	20 (57.1%)	78.5 (8.3)	Not provided	Not provided
	Brolucizumab dose C (4.5 mg), 1 IVT	48	21 (43.8%)	75.2 (7.7)	Not provided	Not provided
	Brolucizumab dose D (6.0 mg), 1 IVT	40	15 (37.5%)	74.5 (9.8)	Not provided	Not provided
Alcon Research^a^ 2016	Aflibercept, 2.0 mg, 8 IVT	45	20 (44.4%)	77.3 (9.1)	55.6 (12.3)	495.7 (144.6)
	Brolucizumab, 6.0 mg, 7 IVT	44	16 (36.4%)	78.8 (9.7)	54.1 (13.9)	490.1 (149.2)
Alcon Research^b^ 2017	Brolucizumab, 3 mg, 3 IVT	25	16 (64.0%)	71.1 (8.53)	Not provided	Not provided
	Brolucizumab, 6 mg, 3 IVT	25	14 (56.0%)	73.6 (7.09)	Not provided	Not provided
Alcon Research^c^ 2019	Aflibercept, 2.0 mg, 10 IVT	360	166 (46.1%)	76.2 (8.80)	Not provided	Not provided
	Brolucizumab, 3 mg, 10 IVT	358	148 (41.3%)	76.7 (8.28)	Not provided	Not provided
	Brolucizumab, 6 mg, 10 IVT	360	155 (43.1%)	76.7 (8.95)	Not provided	Not provided
Alcon Research^d^ 2019	Aflibercept, 2.0 mg, 10 IVT	369	157 (42.5%)	75.5 (7.87)	60.8 (12.93)	Not provided
	Brolucizumab, 6 mg, 10 IVT	370	160 (43.2%)	74.8 (8.58)	61.5 (12.59)	Not provided
Novartis Pharmaceuticals 2020^e^	Brolucizumab, 6 mg, 3 IVT	107	38 (35.5%)	80.6 (8.63)	Not provided	Not provided
	Aflibercept, 2.0 mg, 10 IVT	43	21 (48.8%)	77.9 (9.20)	Not provided	Not provided

a Efficacy and Safety Study of ESBA1008 vs. EYLEA^®^.

b Safety and Pharmacokinetics of RTH258 in Subjects with Age-Related Macular Degeneration.

c Efficacy and Safety of RTH258 vs. Aflibercept—Study 1 (HAWK).

d Efficacy and Safety of RTH258 vs. Aflibercept—Study 2 (HARRIER).

e Study of Safety and Efficacy of Brolucizumab 6 mg Drug Product Intended for Commercialization in Patients with nAMD.

IVT, intravitreal injection.

### 3.3 Estimation of Efficacy

#### 3.3.1 Mean Changes in Best Corrected Visual Acuity

**FIGURE 3:**
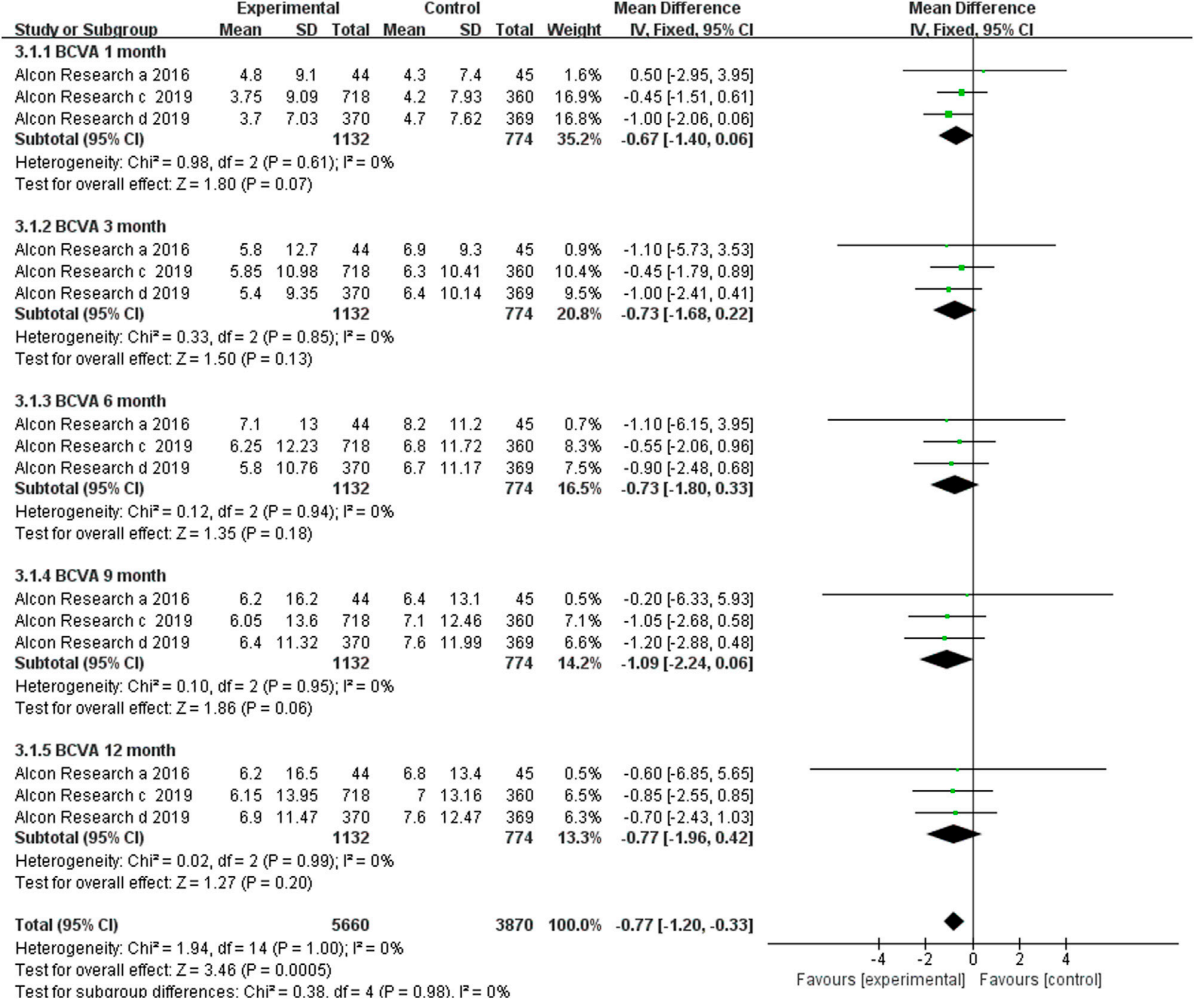
Comparison of the effect on BCVA between the brolucizumab-treated group and control group.

Only three RCTs reported BCVA change from baseline at the time points of 1, 3, 6, 9, and 12 months after the first dosage. In the three included RCTs, brolucizumab with dosages of 3 mg or 6 mg was administered in the experiment group, while its counterpart aflibercept was administered in the control group with a dosage of 2 mg. Our meta-analysis results revealed that no statistically significant difference in the BCVA change from baseline, which was detected at 1 (MD −0.67, 95% CI −1.40 to 0.06, *p* = 0.07), 3 (MD −0.73, 95% CI −1.68 to 0.22, *p* = 0.13), 6 (MD −0.73, 95% CI −1.80 to 0.33, *p* = 0.18), 9 (MD −1.09, 95% CI −2.24 to 0.06, *p* = 0.06), and 12 (MD −0.77, 95% CI −1.96 to 0.42, *p* = 0.20) months after first dosage (Figure 3). According to the research results, brolucizumab was non-inferior to aflibercept in terms of BCVA change efficacy. Moreover, a fix-effects model was used because of low heterogeneity was found between the studies with additive *I* ^2^ = 0%. The funnel plot indicated that there was no publication bias (Supplementary Figure S1).

#### 3.3.2 Significant Reduction in Central Sub-Field Thickness

**FIGURE 4:**
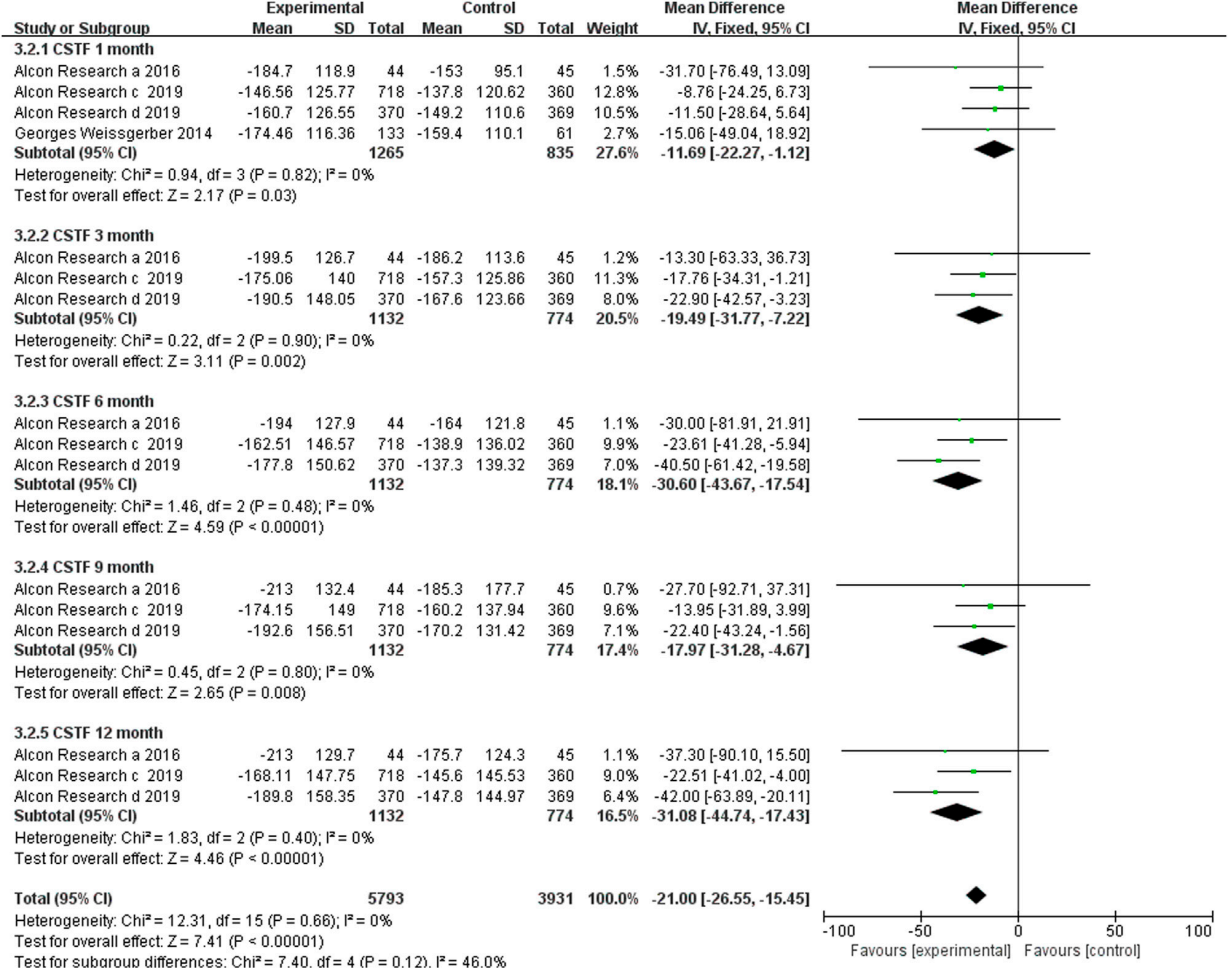
Comparison of the effect on changes in CSFT between the brolucizumab-treated group and control group.

Data from four studies assessed the mean changes in CSFT from the baseline at month 1, whereas three studies reported this parameter at months 3, 6, 9, and 12. The results indicated that the brolucizumab-treated group had higher CSFT reduction than the aflibercept-treated group, and there were significant differences at month 1 (MD −11.69, 95% CI −22.27 to −1.12, *p* = 0.03), 3 (MD −19.49, 95% CI −31.77 to −7.22, *p* = 0.002), 6 (MD −30.60, 95% CI −43.67 to −17.54, *p* < 0.00001), 9 (MD −17.97, 95% CI −31.28 to −4.67, *p* = 0.008), and 12 (MD −31.08, 95% CI −44.74 to −17.43, *p* < 0.00001) months (Figure 4). Low heterogeneity was found in the pooled synthesis with *I* ^2^ = 0%.

### 3.4 Evaluation of Safety

#### 3.4.1 Adverse Events

**FIGURE 5:**
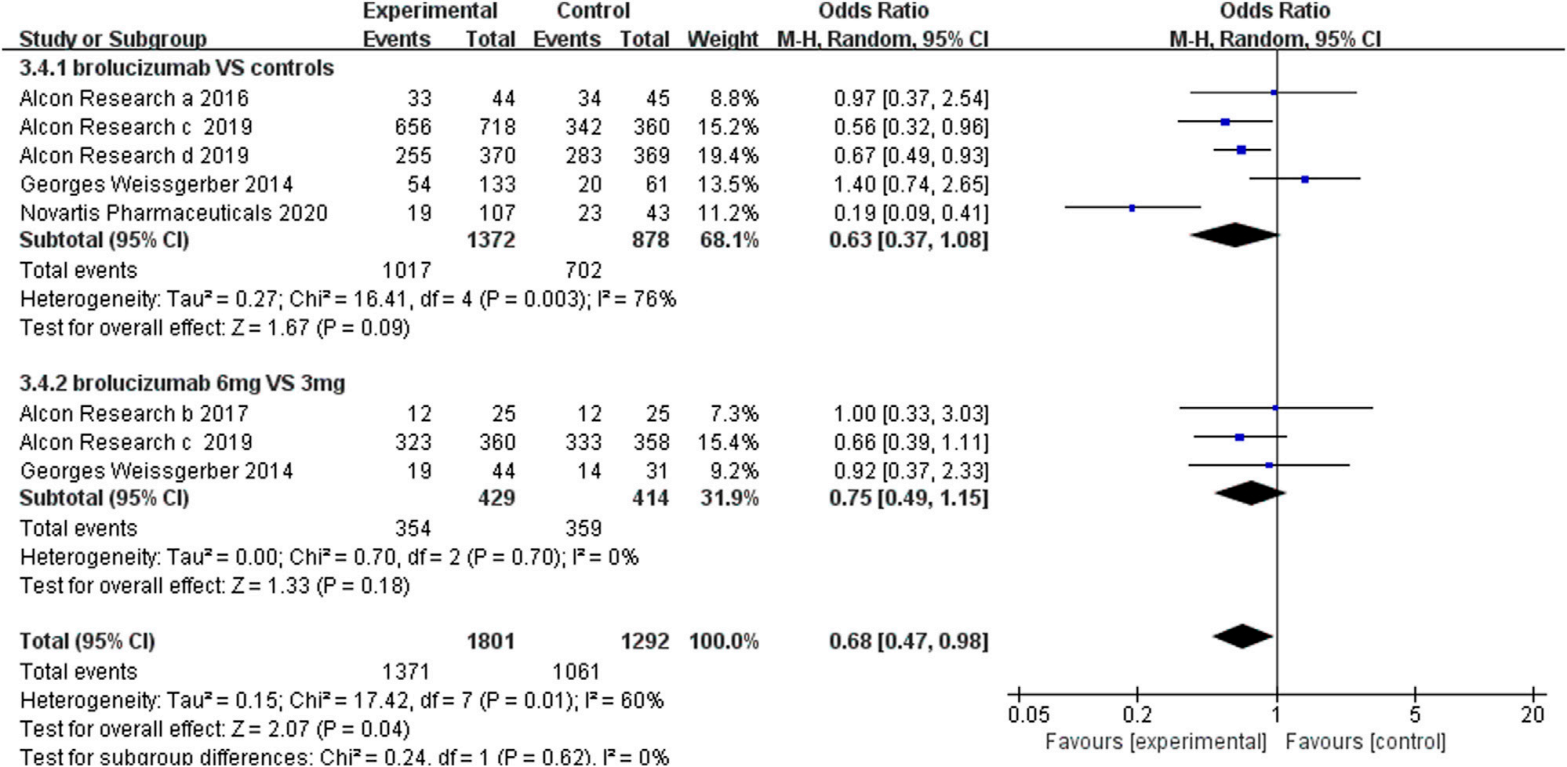
Forest plots of the adverse events.

The incidence of adverse events, severe adverse events, and severe ocular adverse events was used to evaluate the safety of brolucizumab. The analysis of the adverse event incidence from five RCTs noted that there were no obvious differences between the brolucizumab cohort and control cohort (OR 0.63, 95% CI 0.37 to 1.08, *p* = 0.09) (Figure 5). High heterogeneity was detected in the calculation, so sensitivity analysis was performed. After removing the extended experiment (Novartis Pharmaceuticals 2020), the results demonstrated that brolucizumab caused fewer adverse events (OR 0.74, 95% CI 0.58 to 0.94, *p* = 0.01) with low heterogeneity (*I* ^2^ = 46%) (Supplementary Figure S2). Three studies further investigated adverse events of 6 and 3 mg of brolucizumab, results of which showed that the incidence of adverse events of the two dosage groups was comparable (OR 0.75 95% CI 0.49 to 1.15, *p* = 0.18) (Figure 5). Due to the presence of moderate heterogeneity (*I* ^2^ = 60%), a random-effects model was applied for analysis.

#### 3.4.2 Serious Adverse Events

**FIGURE 6:**
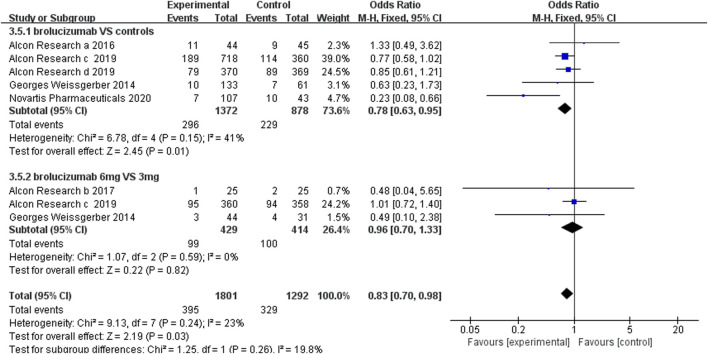
Forest plots of the serious adverse events.

Serious adverse events included cardiac disorders, blood and lymphatic system disorders, hepatobiliary disorders, benign, malignant, and unspecified neoplasms (including cysts and polyps), infections and infestations, nervous system disorders, and vascular disorders. Our results revealed that the number of serious adverse events was less in brolucizumab than controls (OR 0.78, 95% CI 0.63 to 0.95, *p* = 0.01) (Figure 6). Moreover, the number of serious adverse events did not increase with the dosage increment from 3 to 6 mg (OR 0.96, 95% CI 0.70 to 1.33, *p* = 0.82) (Figure 6).

#### 3.4.3 Serious Ocular Adverse Events

**FIGURE 7:**
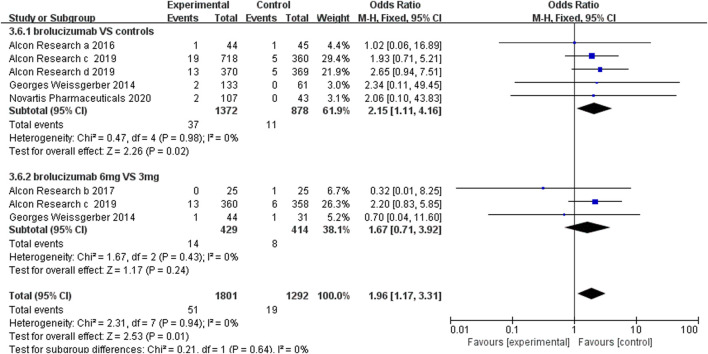
Forest plots of the serious ocular adverse events.

Serious ocular adverse events, such as cataract, conjunctival hemorrhage, eye pain, and nAMD of the other eye, were used to evaluate the safety of brolucizumab separately. The statistical results demonstrated that brolucizumab could lead to more serious ocular adverse events than Lucentis and aflibercept (OR 2.15, 95% CI 1.11 to 4.16, *p* = 0.02) (Figure 7). However, there was no significant difference between the 3-mg brolucizumab and 6-mg brolucizumab groups (OR 1.67, 95% CI 0.71 to 3.92, *p* = 0.24) (Figure 7).

## 4 Discussion

VEGF has been identified as a pivotal factor in promoting the development of choroidal neovascularization. Anti-VEGF treatment for nAMD has been approved for almost a decade with an aim to minimize disease activity. The aim of nAMD treatment was to minimize disease activity. So, changes in BCVA and CSFT from the baseline can be used to evaluate the disease progression and drug efficacy. In this article, synthetic results showed that the effect of brolucizumab on BCVA was not inferior to that of control drugs (Lucentis and aflibercept). In addition, brolucizumab contributed to higher CSFT reduction than aflibercept(2020).

Brolucizumab is a single-chain variable fragment inhibiting the VEGF signal pathway with a molecular weight of approximately 26 kDa. Compared with similar anti-VEGF drugs for the treatment of nAMD, it is much soluble and stable, with a longer injection interval, which can reduce the drug burden of patients (Yu et al., 2021; Ferro Desideri et al., 2021). Studies have demonstrated that the efficacy and safety of brolucizumab is similar to that of aflibercept at up to 12-week injection intervals (Dugel et al., 2020; Dugel et al., 2021). However, the relatively high incidence of intraocular inflammation, particularly retinal vascular inflammation and occlusion of brolucizumab, has raised safety concerns (Motevasseli et al., 2021). Our study will provide stronger evidence for the safety of brolucizumab through summary analysis. Although the incidence of adverse events in brolucizumab was similar to that in the controls, brolucizumab caused more serious ocular adverse events than Lucentis and aflibercept. This result was consistent with existing studies showing that brolucizumab can cause severe intraocular inflammation (Monés et al., 2021; Motevasseli et al., 2021; Nguyen et al., 2021; Khanani et al., 2022).

However, this study has some limitations. Only six RCTs with a small number of participants were involved in this study; thus, the statistical results may deviate from the real world data. The design of the included RCTs varied from each other, with different duration courses and different dosages. For example, Georges et al (2014) was a single-dose vitreous injection study; Alcon Research b, 2017 focused on a pharmacokinetic study with a duration of only 51 days; and Novartis Pharmaceuticals 2020 was an extension study that was a within-patient comparison, which increased heterogeneity among RCTs. Finally, the statistics calculation was too simple with only five parameters. For example, liquid resolution could have been used as a consideration for effectiveness of the indicators. Since the included studies lacked this result, they were not considered. Notwithstanding the aforementioned limitations, it is the first study to perform a meta-analysis of the efficacy and safety of brolucizumab, providing evidence for its curative effect in nAMD.

## 5 Conclusion

Brolucizumab was non-inferior to aflibercept in improving BCVA and induced higher CSFT reduction. The brolucizumab’s incidence of adverse events was similar to that of controls. Although fewer serious adverse events were reported for brolucizumab, it caused more serious ocular adverse events such as cataracts, conjunctival hemorrhage, eye pain, and neovascular age-related macular degeneration of the other eye. In the future, in order to further assess the long-term effectiveness and safety of brolucizumab, more clinical trials with longer research time and more subjects are needed.

## Data Availability

The original contributions presented in the study are included in the article/Supplementary Material, further inquiries can be directed to the corresponding authors.
