# Supplementary figures and images for: The Efficacy and Safety of Brolucizumab for the Treatment of nAMD: A Systematic Review and Meta-Analysis
Source: Front Pharmacol. 2022 May 13;13:890732. doi: 10.3389/fphar.2022.890732 (PMC9136056; doi:10.3389/fphar.2022.890732)

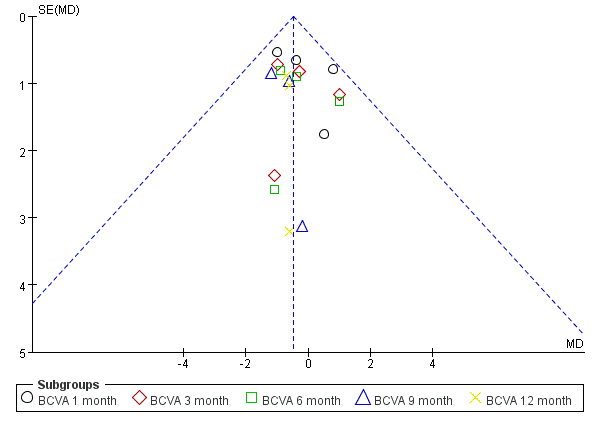

Supplement: Supplementary file 1 [file Image1.JPEG]

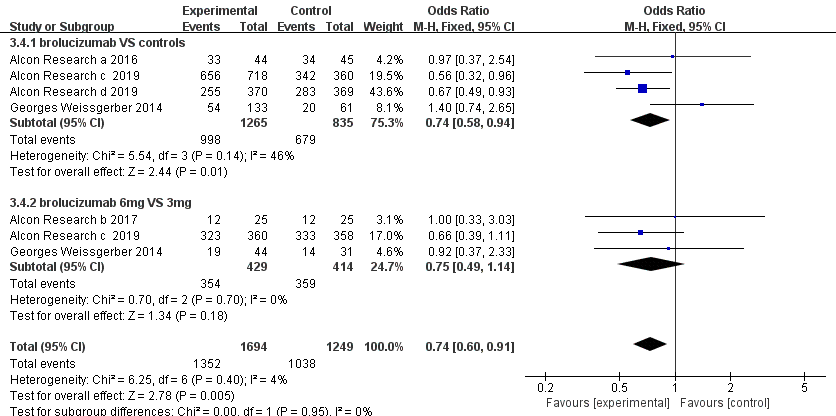

Supplement: Supplementary file 2 [file Image2.JPEG]
