# Supplementary material for: The Efficacy and Safety of Brolucizumab for the Treatment of nAMD: A Systematic Review and Meta-Analysis
Source: Front Pharmacol. 2022 May 13;13:890732. doi: 10.3389/fphar.2022.890732 (PMC9136056; doi:10.3389/fphar.2022.890732)
Supplement: Supplementary file 3 [file DataSheet1.pdf]

Supplementary Figure Captions

Supplementary Figure S1 The funnel plot of BCVA change meta-analysis

Supplementary Figure S2 Forest plots of the adverse events after removing the extended experiment (Novartis Pharmaceuticals 2020)
